# Selectivity of mTOR-Phosphatidic Acid Interactions Is Driven by Acyl Chain Structure and Cholesterol

**DOI:** 10.3390/cells11010119

**Published:** 2021-12-30

**Authors:** Jolanta Żelasko, Aleksander Czogalla

**Affiliations:** Department of Cytobiochemistry, Faculty of Biotechnology, University of Wrocław, F. Joliot-Curie 14a, 50-383 Wroclaw, Poland; jolanta.zegarlinska@uwr.edu.pl

**Keywords:** phosphatidic acid, mTOR, lipid signaling, protein-lipid interactions, BLI, GUV, liposomes, giant unilamellar vesicles, bio-layer interferometry

## Abstract

The need to gain insights into the molecular details of peripheral membrane proteins’ specificity towards phosphatidic acid (PA) is undeniable. The variety of PA species classified in terms of acyl chain length and saturation translates into a complicated, enigmatic network of functional effects that exert a critical influence on cell physiology. As a consequence, numerous studies on the importance of phosphatidic acid in human diseases have been conducted in recent years. One of the key proteins in this context is mTOR, considered to be the most important cellular sensor of essential nutrients while regulating cell proliferation, and which also appears to require PA to build stable and active complexes. Here, we investigated the specific recognition of three physiologically important PA species by the mTOR FRB domain in the presence or absence of cholesterol in targeted membranes. Using a broad range of methods based on model lipid membrane systems, we elucidated how the length and saturation of PA acyl chains influence specific binding of the mTOR FRB domain to the membrane. We also discovered that cholesterol exerts a strong modulatory effect on PA-FRB recognition. Our data provide insight into the molecular details of some physiological effects reported previously and reveal novel mechanisms of fine-tuning the signaling cascades dependent on PA.

## 1. Introduction

Discovering the existence of modulatory effects of membrane physicochemical properties on lipid-dependent signaling pathways makes the latter more complicated and interdependent [1,2]. Thus, the more signaling molecules we identify, the more questions about mechanisms that fine-tune their activity arise. Most recently, the rapid development of lipidomics that led to the identification of a plethora of lipid signaling molecules helped us to understand that even tiny differences in acyl chain composition of membrane phospholipids may dramatically change their physiological functions. An illustrative example is cardiolipin, as saturation of acyl chains switches this lipid from an antagonist to an activator of TLR (toll-like receptor) 4, thus discriminating between pro- and anti-inflammatory responses in macrophages [3]. Increasing evidence points to the possibility that this phenomenon is valid also in the case of other signaling lipids, including phosphatidic acid (PA).

Protein-lipid interactions still remain hard to explore and evaluate. Studying such interactions in the context of living cells is challenging due to their complex architecture and very dynamic nature. On the other hand, using artificial membranes with purified proteins brings a risk of oversimplification of the studied system. A number of studies indicated that some proteins which bind to negatively charged lipids have sequence stretches enriched in basic amino acids [4,5]. Others suggest that certain proteins, e.g., synuclein, ArfGAP1, and melittin, interact with membranes due to their amphipathic motifs [6,7,8]. So far, only in the case of a few membrane lipids, such as phosphoinositides, common sequential or structural lipid-recognition motifs within membrane interacting proteins have been defined [9]. Although for PA dozens of partner proteins involved in various processes in mammalian cells have been identified [10], no common PA-recognizing motif was described for these proteins [11,12,13].

This lack of identified PA-recognition protein domains or patterns is just one of many features that make PA so unique. Despite a low concentration in a cell (around 1% of phospholipids) [14,15,16], PA is central for glycerophospholipid metabolism and cellular signaling [17]. It is implicated in cell proliferation and vesicular trafficking [18] and plays a major role in the local remodeling of membranes [19]. Depending on the local environment (e.g., presence of other lipids and divalent ions, pH at physiological range), the PA head group net charge can be altered [20,21]. It is obvious that all these factors have an impact on recognition of PA-enriched membranes by proteins. Such phenomena have been described for several proteins, including Yas3p [22], Spo20 [23], Opi1 [24] and lipin 1 [25]. For a few PA binding proteins, a cluster of basic, positively charged residues has been described as critical for PA binding [12,26,27]. The uniqueness of PA is nicely described in the hydrogen bond switch model, which has been proposed to explain the mechanism of interaction of the lipid with protein partners [28]. This model relies on the importance of electrostatic bonds between PA and Arg/Lys residues and hydrogen bond formation. As it does not fully resolve specificity towards PA, it may explain the preference of PA recognition over other negatively charged phospholipids in the membrane.

One of the PA-dependent signaling proteins is mTOR (mammalian target of rapamycin). Although some data about mTOR PA recognition are available, the exact mechanism that governs its specificity remains unknown. mTOR is a multidomain kinase that belongs to the PIKK family [29]. This protein has recently been localized to various intracellular compartments including the nucleus and specific components of the endomembrane system such as lysosomes [30]. mTOR is a critical regulator of cell growth in animals and plays an important role in controlling metabolism and proliferation. Aberrations in mTOR-dependent signaling pathways are associated with numerous diseases, e.g., cancer, metabolic disorders, and inflammation [31,32]. mTOR forms two multi-component complexes in the cell: mTORC1 and mTORC2. Each of them is responsible for phosphorylation of a different set of substrates and regulates various processes [31,33,34,35]. Moreover, their activation takes place in certain subcompartments of the cell. As has been shown for mTORC1, activation of this complex is dependent on its recruitment from the cytoplasm to the lysosomal membranes [36,37]. On the other hand, mTORC2 activity has been attributed to the plasma membrane, outer mitochondrial membranes, and endosomal vesicles [38].

Although for decades mTOR has been known to sense amino acids, glucose, and cellular energetics, its interaction with PA was discovered somewhat later [39,40,41]. Numerous in vitro studies on cells have shown that stimulation with exogenous PA or overexpression of PA-producing enzymes can stimulate mTOR signaling [40,42,43,44]. On the other hand, inhibition of mTOR activity has been shown to occur in response to blocking the generation of PA, which was surprising taking into consideration that mTOR activity is regulated by multiple stimuli [45,46,47,48]. Further studies led to the identification of the region within the mTOR protein responsible for PA sensing by both mTOR complexes, namely the FRB domain (FKBP12 rapamycin binding domain) [49,50]. It substantially overlaps with the region to which rapamycin binds, which led to the conclusion that both PA and rapamycin FKBP12 bind mTOR competitively. The latter was demonstrated, inter alia, in human breast cancer cell lines [51].

Within a single cell, there can be found many PA species that differ in fatty acid chain length, saturation level, and as a consequence in cellular function [52]. The type of PA that is formed at a certain time and localization depends on the synthesis pathway and preferential substrates. The latter are mostly lysophosphatidic acid, diacylglycerol, or phosphatidylcholine (PC) [15]. Initially, it was believed that the PA synthesized through PC conversion plays the most significant role as the cellular second messenger [39]. Further research on PA protein partners revealed their altered behavior in response to PA structural differences. For example, among three proteins–Spo20, Opi1, and PDE4A1–significant differences in PA recognition were detected depending on the length and saturation of acyl chains of the lipid. In addition, Spo20 and PDE4A1 preferred mono and bis unsaturated PAs (18:0/18:1 and 16:0/18:2, respectively), while Opi favored polyunsaturated PAs [53]. On the other hand, the activity of the MAPK cascade increased most dramatically in the presence of saturated PA (16:0 PA) [54]. In the case of PA–mTOR interactions, Foster and collaborators proposed that PA with some degree of unsaturation is required for functional activation of mTOR [51]. Indeed, 16:0 PA was shown to inhibit the mTORC2 complex [55] while unsaturated species (16:0/18:1) stimulated both mTORC1 and mTORC2 [50,56]. According to the proposed model, saturated PA species would be directed to energy storage and converted to triacylglycerols, which led to mTOR inhibition, whilst PA with at least one unsaturated bond in acyl chains activates mTOR and the proliferation mode [39,57]. On this basis, further studies revealed the greatest influence on mTORC1 and mTORC2 activity for PAs containing (in decreasing order): oleic (18:1), linoleic (18:2), arachidonic (20:4) acyl chains, while a negative effect was exerted by PA with two palmitic acyl chains (16:0) [50,56].

Although we already know a lot about the physiological consequences of mTOR–PA interactions, a more detailed model explaining, e.g., how membrane context affects protein binding to the lipid and how the differences between molecular species of PA contribute to the mechanism of this interaction is currently not available. Most of the above-mentioned conclusions on these interactions derived from cell-based assays or simplified in vitro binding studies. Therefore, there arose an urgent need to gain deeper insight into PA–mTOR interactions and elucidate molecular mechanisms that govern their specificity. In this report, we describe how a combination of qualitative and quantitative in vitro approaches based on different model membrane systems employed to systematically compare interactions of various PA species embedded in membranes with the recombinant FRB domain of mTOR helped us to explain the impact of the structure of acyl chains of PA on the selectivity and specificity of these interactions. Moreover, we identified cholesterol as a strong modulator of FRB–PA interactions, which seems to be physiologically relevant in light of its function as a major regulator of membrane fluidity and the variability of its content along different cellular subcompartments. The obtained results may help to identify unknown molecular mechanisms of modulation of mTOR activity and its membrane recruitment.

## 2. Materials and Methods

### 2.1. Antibodies, Chemicals, Reagents

2-[4-(2-Hydroxyethyl)piperazin-1-yl]ethane-1-sulfonic acid (HEPES), isopropyl β-d-1-thiogalactopyranoside (IPTG), phenylmethylsulfonyl fluoride (PMSF), sodium dodecyl sulfate (SDS), Tris HCl, and Triton X-100 were purchased from Roth (Karlsruhe, Germany). β-mercaptoethanol, bovine serum albumin (BSA), imidazole, and lysozyme were obtained from Sigma-Aldrich (Schnelldorf, Germany). Bromophenol blue, chloroform, ethylenediaminetetraacetic acid (EDTA), glycerol, and methanol were obtained from POCH (Gliwice, Poland). A cholesterol oxidase/peroxidase kit was purchased from BioSystem (Costa Brava, Spain). cOmplete EDTA-free Protease Inhibitor Cocktail was purchased from Roche (Basel, Switzerland). ECL Prime Western Blotting Detection Reagent was purchased from Amersham, GE Healthcare Life (Chalfont St Giles, UK). Kanamycin was obtained from Bioshop Canada (Burlington, ON, Canada). OMNI nuclease was purchased from EURX (Gdańsk, Poland). Primary antibodies recognizing the FRB domain were purchased from Enzo Life Sciences (Farmingdale, NY, USA) and secondary goat-anti-rabbit antibodies were from Santa Cruz Biotechnology (Dallas, TX, USA). Restriction enzymes BamHI and XhoI were purchased from New England BioLabs (Ipswich, MA, USA). Ribonuclease A (MW = 13.7 Da) and a gel filtration LMW Calibration Kit were obtained from Cytiva (Marlborough, MA, USA). Sodium chloride (NaCl) and Sucrose were purchased from Chempur (Piekary Śląskie, Poland). A Quick Change II Site-Directed Mutagenesis Kit was obtained from Agilent Technologies (Santa Clara, CA, USA). TALON Metal Affinity Resin was purchased from Takara Bio (Kusatsu, Japan).

All the lipids–1,2-dipalmitoyl-sn-glycero-3-phosphate (DPPA), 1,2 dipalmitoyl sn glycero 3 phosphoethanolamine-*N*-(lissamine rhodamine B sulfonyl) (Rh DPPE), 1 palmitoyl 2 oleoyl sn glycero 3 phosphate (POPA), 1 palmitoyl 2 oleoyl glycero 3 phosphocholine (POPC), 1 palmitoyl 2 oleoyl sn glycero 3 phospho L serine (POPS), 1 stearoyl 2 arachidonoyl sn glycero 3 phosphate (SAPA), and cholesterol (CH) were purchased from Avanti Polar Lipids (Alabaster, AL, USA)

### 2.2. DNA Constructs

The pGEX-2T plasmid containing the sequence for the FRB domain of human mTOR protein was bought from Addgene (Cambridge, MA, USA) (cat. no. 26607). The FRB gene sequence (encoding aa 2015-2114) (GenBank ID: NM_004958) was subcloned into the pET28 plasmid with the 8His tag (kindly provided by Magdalena Zaremba-Czogalla, University of Wrocław) using BamHI and XhoI restriction enzymes to generate the expression vector pET28 8His FRB. One single insertion was required to remove the STOP codon and to change the reading frame of the above-mentioned vector to obtain the vector pET28 8His FRB-mGFP (achieved with Quick Change II Site-Directed Mutagenesis Kit (Agilent) and the primers 5′CGACGAATCTCAAAGCAGGCTAGAATTCTGGAAGTGC and 5′GCACTTCCAGAATTCTAGCCTGCTTTGAGATTCGTCG). A construct with mGFP protein was used for giant unilamellar vesicle (GUV) experiments. The control vector mGFP-C1was purchased from Addgene (cat. no. 54759).

### 2.3. Expression and Purification of Recombinant Proteins

Production of recombinant proteins, FRB and FRB-GFP and GFP, was carried out in Escherichia coli NiCo21 (DE3). Initially, cells were precultured overnight at 37 °C under constant agitation (200 rpm) in 5 mL of LB medium containing kanamycin (35 µg/mL). Then, 200 mL of fresh LB medium with kanamycin (35 μg/mL) was inoculated with overnight preculture and grown at 37 °C to reach optical density (OD600) of 0.7, and IPTG (final concentration 0.75 mM) was added to induce protein overexpression and incubated for another 16–18 h at 18 °C with constant shaking (200 rpm). Bacteria were then centrifuged (20 min, 4 °C, 10,000× *g*) and the bacterial lysate was prepared by resuspending the pellet in lysis buffer (10 mM HEPES, 500 mM NaCl, 1mM PMSF, 10 mM imidazole, 0.1% Triton X-100, 25 U/mL OMNI nuclease, 1 mg/mL lysozyme, cOmplete EDTA-free Protease Inhibitor Cocktail, pH 7.4); thereafter it was incubated on ice for 30 min, sonicated (Hielscher sonicator) 10 times for 15 s with 15 s intervals on ice and centrifuged (35,000× *g*, 30 min, 4 °C). The obtained supernatant was incubated for 2 h at 4 °C with 1 mL bed volume of TALON Metal Affinity Resin with gentle rotation. The resin was in advance pre-equilibrated with the lysis buffer. Beads were packed into the column, washed with 20 mL of wash buffer 1 (10 mM HEPES, 500 mM NaCl, 10 mM imidazole, pH 8.0), wash buffer 2 (10 mM HEPES, 300 mM NaCl, 10 mM imidazole, pH 8.0), and wash buffer 3 (10 mM HEPES, 300 mM NaCl, pH 8.0) until absorbance A280 was lower than 0.01. Elution was performed with elution buffer (10 mM HEPES, 300 mM NaCl, 200 mM imidazole, pH 8.0). Concentrations of FRB, FRB GFP, and GFP were determined by UV absorption spectroscopy using a Cary 1E spectrophotometer at λ = 280 nm employing extinction coefficient parameters determined using the ProtParam tool (https://web.expasy.org/protparam/, accessed on 28 February 2021) [58]. Stepwise dialysis of purified protein to buffers (I: 10 mM HEPES, 250 mM NaCl, 20 mM EDTA, 1 mM DTT; II: 10 mM HEPES, 200 mM NaCl, 1 mM DTT; III: 10 mM HEPES, 150 mM NaCl, 1 mM DTT; pH 7.4 for all buffers) was performed using membrane Spectra/Por 6 pre-wetted with cut-off 10 and 25 kDa (for FRB, GFP, and FRB GFP, respectively) (Spectrum Chemical, New Brunswick, NJ, USA). Prior to use, the purified proteins were centrifuged for 10 min at 896× *g* and 4 °C to remove any precipitated material. In order to assess the quality of the purified proteins, they were mixed with 5x reducing protein sample buffer (50 mM Tris-HCl pH 8.0, 12.5% SDS, 0.15% bromophenol blue, 5 mM EDTA, 20% glycerol, 2.5% β mercaptoethanol), boiled for 10 min at 95 °C and subjected to SDS-PAGE. Other quality check procedures are described in Supplementary Information.

### 2.4. Preparation of Large Unilamellar Vesicles (LUVs)

Concentrations of lipids dissolved in organic solvents were quantified by total phosphorus analysis [59]. Cholesterol concentration was determined using a BioSystems Cholesterol Kit according to the manufacturer’s protocol. Lipid mixtures were prepared according to molar ratios of 90 mol% POPC and 10 mol% PA or 55 mol% POPC, 35 mol% CH, and 10 mol% PA. For LUV preparation, lipid mixtures in chloroform (for DPPA chloroform/methanol) were dried in a round-bottom flask under a nitrogen gas stream, followed by incubation under a vacuum desiccator for at least 2 h, or overnight to remove traces of organic solvents. The dried lipid films were rehydrated with SLB buffer (10 mM HEPES, 150 mM NaCl, pH 7.4) to a final lipid concentration of 1 mg/mL. Multilamellar liposomes were then subjected to 5 cycles of freezing in liquid nitrogen and thawing (room temperature). Vesicles suspension was sequentially extruded through a polycarbonate membrane filter with pore sizes (diameter) 0.2 µm and 0.1 µm (Whatman Nuclepore, Fisher Scientific, Hampton, NH, USA) under high-pressure gaseous nitrogen in a temperature above the phase transition of each PA. Each extrusion step was repeated 11 times (cycles). Liposomes were snap-frozen in liquid nitrogen and stored at −80 °C until use. For quality control, prior to use in binding assays, the size and zeta potential of the liposomes were determined using a ZetaSizer Nano ZS (Malvern Instruments, Malvern, UK). Procedures are described in detail in Supplementary Information.

### 2.5. Flotation Assay

The binding of proteins to liposomes was determined via flotation assay using ultracentrifugation in a step density gradient of sucrose in SLB buffer. FRB at concentration 360 µg/mL (25 µM) was mixed with 0.4 mg/mL LUVs to a final volume of 250 µL and incubated for 30 min at room temperature. Control samples contained FRB at the same concentration but without liposomes. After incubation, samples were mixed with 0.25 mL of 60% sucrose to reach the final concentration of 30% and the step gradient was built on top of it (in sequence: 0.8 mL 15%, 1.8 mL 10%, and 1 mL 0%). The samples were centrifuged at ~200,000× *g* (45,000 rpm, ultracentrifuge Optima L90K, SW-60 Ti rotor (Beckman Coulter, Brea, CA, USA)) for 2 h at 4 °C. From each tube, six individual fractions were gently collected, starting from the top of the tube, and mixed with SDS to obtain the final concentration of 0.2%. Fractions were analyzed via dot-blot assay. Equal volumes of samples were loaded into the wells of a Dot Blotter (Hoefer Inc., Holliston, MA, USA) and transferred onto a nitrocellulose membrane (Amersham, Protran, UK) using a vacuum pump. The membrane was subsequently blocked with 5% (*w*/*v*) milk (powder) in TBS-T (20 mM Tris-HCl, 150 mM NaCl, and 0.05% Tween 20, pH 7.4) overnight at 4 °C. Incubation with primary antibodies diluted 1:1000 in TBS-T was applied for 3 h at room temperature or overnight at 4 °C. The membrane was washed three times in TBS-T (5 min each). The secondary antibodies diluted 1:10,000 in TBS-T were used for incubation for 1 h at room temperature and subsequently washed as previously. Enhanced chemiluminescence (ECL Prime Western Blotting Detection Reagent, Amersham, UK) was used for detection using BioSpectrum Imaging System (UVP, Upland, CA, USA), in chemiluminescence mode. Image quantification was carried out by 2D densitometry (ImageJ, 1.53c version). The bound fraction of protein was determined as the amount of protein in the second fraction where liposomes floated and was calculated as the amount of protein in this fraction divided by the total protein content in all fractions.

### 2.6. Preparation of Giant Unilamellar Vesicles (GUVs)

GUVs of lipid composition corresponding to LUVs and the addition of 0.1 mol% of Rh-PE were prepared by electroformation (EF) [60,61] or passive formation (PF) [62,63]. In both cases, lipids were mixed in chloroform. For EF 3 µL of a 1 mg/mL lipid solution were deposited on each of the two platinum wires of a custom-made chamber and dried under gaseous nitrogen followed by incubation under a vacuum desiccator for at least 2 h. Sucrose-containing swelling buffer (280 mM Sucrose, 10 mM HEPES, pH 7.4) isosmotic to SLB buffer (310 mOsm/kg), was added to a Teflon chamber, and platinum wires were immersed in it. Chambers were connected to a function generator (NDN DF1641A, NDN Instrument) and exposed to a 2 V (rms) AC electric field for 2 h (first 1.5 h 10 Hz, next 30 min 2 Hz). For PF, dry lipid films formed in round-bottom glass tubes were immersed in swelling buffer and kept overnight in the dark without shaking at room temperature.

### 2.7. GUV Binding Assay and Image Quantification

In order to visualize protein–vesicle interactions, GUVs were mixed with FRB-GFP protein at 1 µM concentration in SLB buffer (for the control experiments GFP was used at the same concentration). A black, 96-well glass-bottom plate (Greiner Bio-One, Kremsmünster, Austria) was blocked with 2% BSA for 20 min, and subsequently washed with SLB buffer. Vesicles with protein were transferred into wells by gentle pipetting and equilibrated for 10 min prior to imaging.

Confocal fluorescence microscopy was carried out on the LSM 510 Meta system (Carl Zeiss, Jena, Germany). GFP fluorescence was excited with a 488 nm argon laser and detected using a BP 505–530 nm filter. Rhodamine fluorescence was excited with a 561 nm DPSS laser and detected using an LP 575 nm filter. Images were analyzed with the Circle Skinner plugin for Fiji (https://sites.imagej.net/CircleSkinner/, accessed on 2 April 2019). GUV identification was verified manually for every confocal image and corrected when necessary.

### 2.8. BLI Measurements

The Octet K2 2-channel System (ForteBio, Menlo Park, CA, USA) was used for kinetics studies. This method allows one to analyze kinetic interactions in real time [64]. Experiments were performed in black, 96-well plates (Nunc F96 MicroWell Plates, Thermo Fisher Scientific, Langenselbold, Germany). The total volume of each sample or buffer was 0.2 mL per well. The test was performed at 30 °C with 1000 rpm shaking. Prior to each assay, Ni-NTA biosensor tips (ForteBio, Menlo Park, CA, USA) were pre-wet in 0.2 mL of SLB buffer for at least 10 min. Subsequently, an equilibration step with SLB buffer was performed for 60 s, after which Ni-NTA biosensor tips were non-covalently loaded with the FRB domain for 500 s, followed by an additional equilibration step (200 s) in SLB buffer. The association step of the FRB domain with LUVs of corresponding composition in the range 0–100 µM of PA was carried out for 500 s. Dissociation was assayed for 600 s. All measurements were performed in triplicate.

### 2.9. BLI Data Analysis

To analyze binding kinetics based on association and dissociation curves, baseline correction was performed. Global fitting for each dataset (covering the whole range of analyzed PA concentrations) was used and the binding model was chosen depending on the PA species in liposomes. Models were used to calculate the association rate constant (ka), defined as the rate of complex formation per second in a 1 molar solution of two reaction partners. To estimate the stability of the complexes the dissociation rate constant (kd) was calculated. The ratio kd/ka gives us the affinity constant KD. The fact that a kd can only be calculated when signal decay greater than or equal to 5% is observed can be considered a drawback in the case of KD estimation [64,65]. Hence, the kd (s−1) is given by kd < −ln(0.95)/td, where td is dissociation time in seconds. All the calculations were performed using Octet Data Analysis High Throughput (HT) software version 11.1.0.25 (ForteBio).

## 3. Results

### 3.1. Structure of Acyl Chains of PA Determines Binding Specificity of mTOR FRB Domain

**Figure 1:**
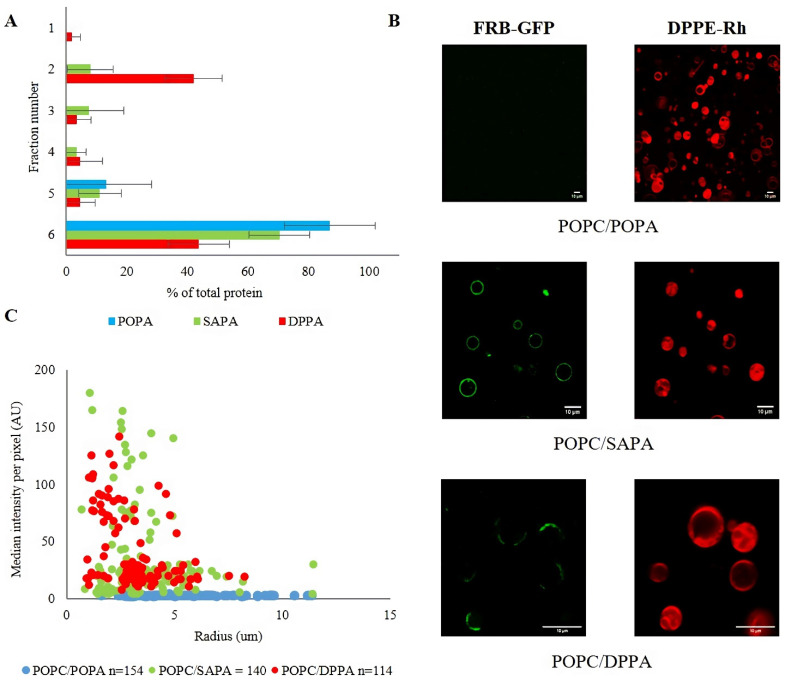
Binding preferences of mTOR FRB domain to different PAs vary in terms of acyl chain structure. Experiments were performed using POPC liposomes containing 10 mol% POPA, SAPA, or DPPA. (**A**) Percentage of FRB domain of mTOR in the fractions collected after flotation and analyzed by dot-blot/densitometry. For POPA and SAPA, no or very weak binding occurs, but for DPPA strong interaction is observed. Error bars are standard deviations of three independent experiments. (**B**) Distribution of median intensity per pixel of individual GUVs (of variable diameter) incubated with FRB-GFP in green fluorescent channel. GUVs were captured after addition of FRB GFP protein and Rh-PE as membrane marker (data from three independent sets of experiments). Each dot represents a single GUV. The number (*n*) of analyzed GUVs is indicated in the legend. (**C**) Representative images of GUVs (with Rh-PE as membrane marker in red) and FRB-GFP (green channel). The scale bar corresponds to 10 μm.

To characterize the capacity of the FRB domain to bind PA-enriched membranes, we focused on phosphatidic acid molecules differing in the acyl chain structure. The diversity in PA structure results from different synthetic pathways within a cell. Depending on the preferred substrates for PA synthesizing enzymes, the product may vary in saturation and length of acyl chains [15]. Previous studies revealed that mTOR activity in cells in vitro is dependent on PA and that the consequences of this interaction are determined by the structure of the lipid [39,40,41]. For example, PA is required firstly for the assembly of mTORC1 and mTORC2 complexes and secondly to activate downstream pathways [50]. Furthermore, it has been suggested that saturated PA inhibits mTORC2 activity, whereas PA with unsaturated acyl chains promotes its signaling [57]. Based on the fact that the PA interacting site of mTOR is localized within its FRB domain [49], we hypothesized that the FRB PA binding might depend on the length and saturation of acyl chains of the lipid. To test our hypothesis, we decided to use three diverse PA species which are of major importance for eukaryotic cells and were chosen based on literature data [55,66,67]. Comparison of DPPA (16:0) with POPA (16:0–18:1) would let us test the importance of chain saturation for binding. As SAPA (18:0–20:4) has longer and more unsaturated acyl chains, comparing it to POPA would allow us to verify whether this corresponds to a larger fraction of exposed acyl chains [68] and whether the size of the cross-sectional area influences the binding. Thus, liposomes used in our studies were composed of POPA, DPPA, or SAPA with POPC (molar ratio 1/9), as the latter lipid is the most abundant PC species in animal cells [69,70]. For the quality control and physicochemical characterization, all LUV preparations were first subjected to thin-layer chromatography (TLC), dynamic light scattering (DLS), and electrophoretic light scattering (ELS) measurements (Figures S1 and S2). The average diameter of liposomes in the range of 100–125 nm and the polydispersity index (PDI) below 0.1 suggest that the particles in suspension are close to homogeneity. Strongly negative values of zeta potential and TLC chromatograms confirmed the presence of PA in vesicles. Regarding the recombinant FRB domain of mTOR, SDS-PAGE and size-exclusion chromatography confirmed that the protein was pure and monomeric in solution, and CD analysis confirmed that it is properly folded and stable (Figure S3). In the flotation assay for liposomes containing PA with unsaturated acyl chains, we observed no (POPA) or weak (SAPA) binding of the FRB domain, which was in contradiction to robust protein recruitment by vesicles containing saturated phosphatidic acid (DPPA) (Figure 1A).

In order to supplement the above results and eliminate potential pitfalls related to the flotation method (effect of membrane curvature, nonequilibrium binding conditions, etc.) [1], we additionally examined the binding of the FRB domain fused to GFP (FRB-GFP, see Figure S3) to GUVs. Among artificial model membranes, GUVs are among the most appropriate and most widely used tools due to their nearly flat geometry of the freestanding lipid bilayer [71,72]. In this case, we used the same molar ratios of lipids as for LUVs, although for visualization 0.1 mol% Rh-PE was added. As shown in confocal images (Figure 1C), FRB GFP interacted to various degrees with PA-containing vesicles. For GUVs containing POPA, we did not observe any interaction, similarly to GUVs made of pure POPC, which is consistent with results obtained with the flotation method. On the other hand, the binding strength to SAPA was very similar to DPPA. Thus, it seems that there are some intrinsic features characteristic for each of the two vesicle systems and experimental conditions, which result in tiny differences in terms of FRB binding (Figure 1 and Figure S5A), although it cannot be excluded that fusion with GFP slightly alter the behavior of the FRB domain. Nevertheless, the results obtained with both vesicle systems are highly consistent. In control experiments with GFP, we did not observe any binding for any of the lipid compositions tested (Figure S6).

Length and saturation of acyl chains may alter PA accessibility in the context of lipid bilayers. Kulig et al. observed a significant effect of the chain saturation on PC/PA miscibility [73]. According to their results, a system composed of saturated and unsaturated lipids (DOPC/DPPA) displayed complete immiscibility. This is in contrast to systems composed of unsaturated species of both lipids, which showed almost ideal mixing. These observations are in agreement with studies of Cambrea [73,74]. Our results seem to reflect these phenomena at the level of protein-lipid binding. Namely, imaging DPPA-containing vesicles by confocal microscopy revealed a very interesting effect not observed for GUVs containing unsaturated PA species. In the latter case, bound FRB was distributed equally around vesicles, while for DPPA we noticed clusters of the protein on the surface of GUVs. It seems that DPPA as a saturated lipid forms gel domains. It has been reported that the headgroup of POPA in the POPC/POPA system is located deeper than the PC headgroup [73]. Our recently published data obtained for monolayers show bigger APL (area per lipid) for POPC/SAPA mixture (44.6 ± 2.1 Å2) than for POPC/POPA (38.6 ± 2.3 Å2), although in both cases the values are significantly lower than those expected from theoretical calculations [68]. Such disproportion in APL is explained by stronger interaction between POPC and POPA than for POPC and SAPA. It may explain the lack of binding for the POPC/POPA system observed by us. Figuratively speaking, POPA seems to be captured and imprisoned by POPC, being invisible for the protein. For SAPA, we observed a much weaker affinity towards POPC, which is reflected in a weak interaction with mTOR FRB presented in the flotation method and confocal microscopy.

### 3.2. Effect of Cholesterol on FRB-PA Binding

**Figure 2:**
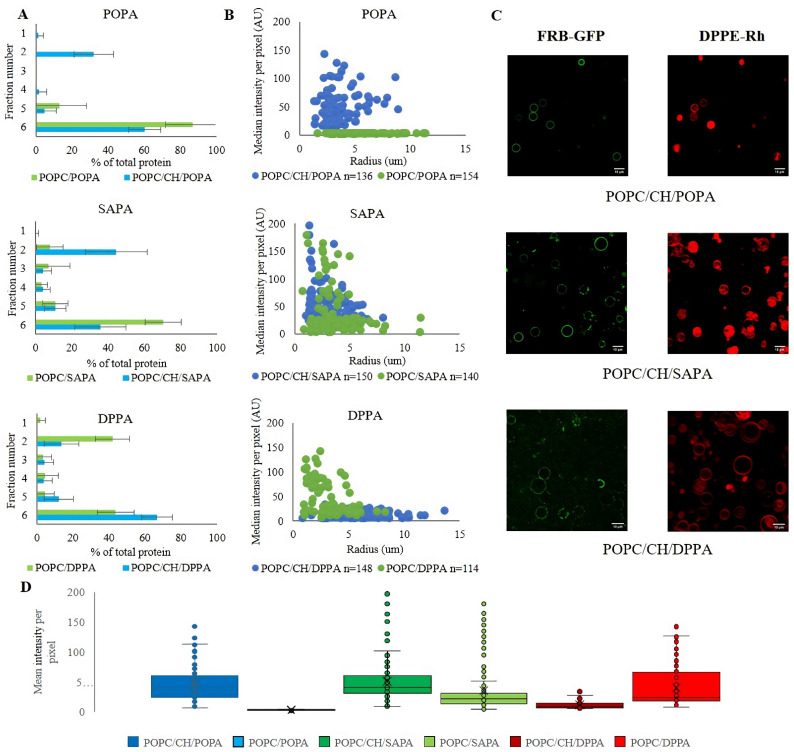
Effect of cholesterol on binding preferences of mTOR FRB domain towards different PA species. Experiments were performed using POPC/CH/PA (55/35/10 molar ratio) liposomes with POPA, SAPA, or DPPA, and results were directly compared to the data already presented in Figure 1. (**A**) Percentage of FRB domain of mTOR protein in fractions collected after flotation and analyzed by dot-blot/densitometry. Error bars are standard deviations of three independent experiments. (**B**) Distribution of median intensity per pixel of individual GUVs incubated with FRB-GFP in green fluorescent channel. Each dot represents a single GUV (with CH represented by blue dots, and without CH by green). Data collected in three independent experiments. The number (*n*) of analyzed GUVs is indicated in the legend. (**C)** Representative images of GUVs (with Rh-PE as membrane marker in red channel) and FRB GFP (green channel). The scale bar corresponds to 10 μm. (**D**) Boxplot of data presented in panel B. Each dot represents a single GUV. The mean value for each GUV lipid composition tested is indicated as a line and standard deviation as whiskers.

Cholesterol is a major regulator of membrane fluidity, permeability, formation of microdomains, and many other physicochemical parameters [75,76,77,78,79]. Studies showed that CH has a great impact on the presentation of phospholipids in the membranes, which is reflected through changes in affinities of peripheral membrane proteins to these lipids [80,81,82]. As CH has a critical influence on multiple parameters of membranes, and as a consequence of the presentation of signaling lipids at the surface of membranes [2], animal cells invest a lot of energy to fine-tune the proper distribution of this sterol, which is reflected by its steep gradient along the secretory pathway [83,84,85]. Therefore, we decided to investigate the influence of CH on the binding of FRB to PA-containing vesicles. We used a CH concentration equal to 35 mol% as it roughly corresponds to the concentration in cellular plasma membranes and endocytic vesicles [86]. First, we performed flotation experiments using LUVs composed of POPC/CH/PA (molar ratio 55/35/10) using the same PA species as above. The addition of CH resulted in dramatic changes in FRB binding in the case of all three examined PAs (Figure 2 and Figure S4A). For unsaturated POPA and SAPA presence of CH increased the percent of protein floating together with liposomal fractions. Opposite behavior was observed for saturated DPPA, where cholesterol diminished binding of FRB. Moreover, in control experiments using POPC/CH vesicles, we found that the FRB domain has no affinity to CH (Figure S4B), thus confirming that cholesterol does not trigger the binding but rather has a capability to modulate it. To prove that the modulatory effect of CH is not related solely to the presence of net charge in the bilayer, we tested the binding of FRB to the most abundant negatively charged membrane lipid, namely PS with or without cholesterol. We observed relatively weak binding in both cases, indicating that the presence of the charge itself is not sufficient for these interactions to occur and PA is indeed required (Figure S4). This is in agreement with other research on lipid selectivity of mTOR [45] but, to some extent, in contradiction to the conclusions drawn by Rodriguez Camargo et al. [87], who reported the lack of specificity of the FRB domain. However, while employing the more systematic approach presented here, which is based on freestanding lipid bilayers, we observed that this interaction is strongly dependent on the acyl chain structure of PA and CH content. Our data also strongly suggest that stabilization of FRB–PA interaction might occur through the electrostatic/hydrogen bond switch mechanism which is not valid for PS. The electrostatic/hydrogen bond switch mechanism occurs when a hydrogen bond between positively charged residue of Arg or Lys with the PA headgroup is created. This causes deprotonation of the PA headgroup (increasing charge from −1 to −2), thereby further stabilizing the interaction [28]. High ionic strength (500 mM NaCl) reduces the affinity of the FRB domain to PA, which supports the electrostatic character of this interaction [49]. The hydrogen bond switch mechanism as a requirement for the occurrence of PA protein interaction has also been reported for other PA binding partners [88,89]. 

The influence of cholesterol on FRB–PA interactions was also visible in our confocal imaging approach. Control experiments with POPC/CH vesicles gave similar results as in the flotation method. Likewise, in the case of GFP protein, we did not observe any binding (Figures S5 and S6). In the case of POPA containing GUVs, CH significantly increased the binding of the protein, whereas for DPPA it acted in an opposite way, which is in full agreement with the data obtained via the flotation method. In the case of GUVs with SAPA, we observed relatively enhanced protein binding compared to LUVs, although the amplitude of the CH-driven modulation is moderate. Previous studies have shown that cholesterol insertion into a phospholipid bilayer makes a highly ordered bilayer less ordered and vice versa [90]. It, therefore, seems quite understandable that for POPC/POPA addition of cholesterol remodeled binding completely. Namely, the introduction of cholesterol could expose the POPA headgroup, release it from interaction with POPC and thus make it more accessible for the FRB domain. The results obtained with SAPA are in agreement with this, as binding was also slightly enhanced by cholesterol insertion. It can be stated that the presence of CH limits PC–unsaturated PA interactions. Considering our recently published data, it is also reflected by the increase in APL values and monolayer compressibility for POPC/CH/POPA and POPC/CH/SAPA, in contrast to corresponding systems without CH. Such a modulatory effect of cholesterol on POPC/POPA and POPC/SAPA systems also confirms the values of Gibb’s free energy of mixing, which is lower in the presence of CH [68]. DPPA in our experimental conditions exists in the gel phase, which is manifested as FRB-GFP-enriched domains observed in confocal microscopy of POPC/DPPA GUVs. We previously observed that the presence of cholesterol in POPC/DPPA mixtures altered membrane characteristics and parameters, i.e., increase in APL and of the gel phase disorder and reorientation of lipids in the membrane. These data indicate the existence of strong DPPA/CH interactions and an increase in DPPA acyl chain exposure induced by CH. It could be that due to all these changes we observe higher and more uniformly distributed binding of mTOR FRB on the surface of POPC/CH/DPPA GUVs compared to POPC/DPPA.

### 3.3. Kinetic Analysis of FRB–PA Interaction by BLI Method

**Figure 3:**
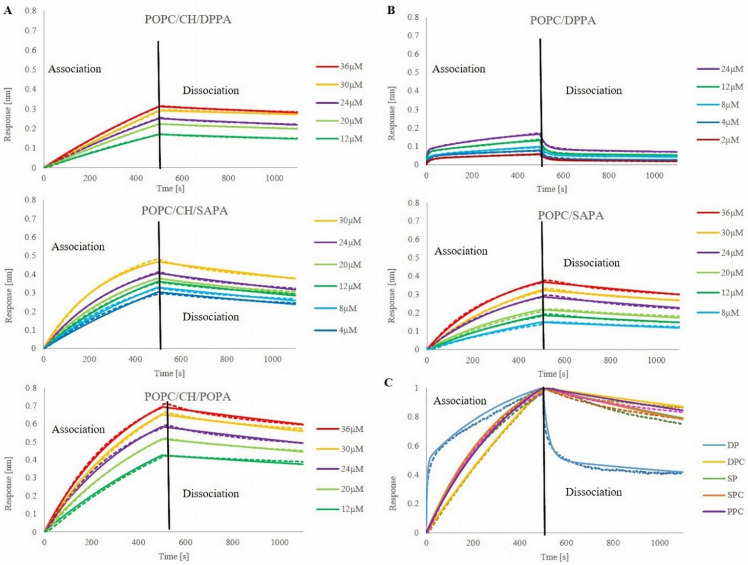
Association and dissociation curves of PA-containing liposomes. mTOR FRB domain was immobilized on Ni-NTA sensors tips prior association. Curves represent the mean values of triplicate measurements of each concentration. Dashed lines represent raw data, continuous lines fit. (**A**) POPC/CH/PA liposomes, (**B**) POPC/PA liposomes, (**C**) normalized curves of all studied liposome mixtures in 24 µM PA concentration.

One method which allows one to measure binding kinetics is bio-layer interferometry (BLI), which, along with surface plasmon resonance (SPR), belongs to the label-free biosensor technologies [91]. BLI technology developed some features that gave it an advantage over the SPR, namely no risk of clogging and the possibility of using crude samples due to the open shaking micro-well plate format; recoverable samples; substantial extension of the association step; a wide range of disposable optical fiber biosensors and high-throughput 96-well and 384-well microplate format [92,93]. Our approach was based on immobilization of the protein via histidine tag to Ni-NTA biosensors in a reproducible manner with standardized buffer conditions and further titration with PA containing liposomes that showed no substantial non-specific binding (Figure S8). After testing the most appropriate concentration of protein, loading (t = 550 s) on biosensor tips was set to 5 µg/mL (Figure S7). As liposomes bind to the FRB-loaded surface, the thickness (nm) of the biosensor layer increased, which was measured as spectral shift (Δλ). It is important to point out that, because of the large size of liposomes, our raw data exhibit a shift of negative value and were flipped prior to data processing. At first, different concentrations of PA present in liposomes were tested for each set of liposomes. Unfortunately, kinetic curves for concentrations around 0.1x KD were unachievable due to the low system response. On the other hand, for PA concentrations around 10x KD curves were collapsing and were not appropriate for further analysis. Thus, for each liposomal formulation, the concentration range was adjusted individually, but in the approximate range (Figure 3). The inability to use the same range of concentrations for all lipid mixtures may arise from the technical problems caused by liposomes. Namely, the relatively large size of the vesicles, their undefined behavior on the sensors, high probability of fusion, aggregation, and/or collapsing may be the source of such problems. Another matter is the distinct characteristics of studied PA species, e.g., DPPA is more rigid and ordered, which also may influence liposomes’ features and behavior.

The lowest measurable concentration of PA was 2 µM (POPC/DPPA) and the highest was 36 µM (POPC/CH/POPA; POPC/SAPA; POPC/CH/DPPA). The highest amplitude of system response was obtained for POPC/CH/POPA liposomes (0.69 nm for 36 µM), in contrast to curves representing the same PA concentration in POPC/SAPA and POPC/CH/DPPA vesicles where 0.36 and 0.31 nm shift could be observed, respectively. The individual association and dissociation curves are presented in Figure 3, while Table 1 displays obtained association rate constants (Ka), dissociation rate constants (Kd), and calculated equilibrium dissociation constants (affinity constants) (KD) values for PA in formulated liposomes. From the presented results, the first notable observation is that liposomes interact with mTOR FBR not only in a concentration-dependent but also in a lipid composition-dependent manner. The measured association rate (Table 1) ranks tested liposomes as follows: POPC/CH/SAPA > POPC/CH/POPA > POPC/SAPA > POPC/CH/DPPA, yet all of them are in an approximate range from 1.13 × 10^2^ to 1.96 × 10^1^ (1/Ms). Notwithstanding, POPC/DPPA exhibits two Ka values, and the first one is in the above-mentioned range, but the second one exhibits a relatively high value equal to 1.15 × 10^4^ (1/Ms). In this case, the 2:1 binding model had a better fit, and for all other mixtures, we followed fitting with the binding model 1:1, which is recognizable by the gradual monophasic rise with a plateau after reaching saturation and equilibrium. Model 2:1 is exploited for nonideal heterogeneous binding with a quick initial rise in signal followed by a slower rate of binding and initial fast dissociation continued with a slower off-rate [94] (see Figure 3C). Values of the dissociation rate constant indicate that while interacting with mTOR FRB PA creates stable complexes with the exception of one of the two values of Kd obtained for POPC/DPPA, where a fast association is followed by rapid dissociation. Because the affinity constant (KD) is the ratio of Kd/Ka, calculated values are undifferentiated, in the same order of magnitude. Although the obtained values are in the proximity range, the trend for POPA and SAPA is clear, where CH acts as a binding modulator (see Figure S8, where POPC/POPA liposomes were tested and showed no binding). This points out how important it is to look at data from different perspectives and try to identify correlations and connections that drive studied processes. If one were to try to explain PA-FRB kinetics based on KD, one might say that it is not influenced by the structural features of PA acyl chains. However, the truth is that at the molecular level, dependencies that determine the processes are not one-factorial. To discover the mechanisms that govern them, one has to pay attention to the details. Although so far, the BLI technique has been used for protein-lipid binding only by one research group [64,95], we believe that, along with our results, it demonstrates that it may be a promising tool to measure such interactions in a quantitative way.

## 4. Conclusions

In this study, we addressed the question of how mTOR, a protein of pivotal importance in controlling cellular metabolism and proliferation, is regulated by phosphatidic acid. The latter is a precursor for the majority of cellular glycerophospholipids, regulates numerous signaling pathways, and is able, due to its molecular shape, to modulate membrane mechanical properties. As cell-based studies show that mTOR-dependent signaling is sensitive in regard to various molecular species of the lipid, it appeared necessary to decipher the molecular mechanisms behind such selectivity. That is why we focused on studying the specificity of the FRB domain, which has been previously recognized as a PA binding site within mTOR, towards biologically relevant PA species. These species differing in acyl chain saturation and length, namely DPPA, POPA, and SAPA, were embedded into highly controllable membrane model systems. Our research demonstrated that the FRB domain binds to PA-enriched membranes, but the degree of acyl chain saturation of PA defines these interactions. Thus, it transpired that although PA is already known as a factor that governs mTOR’s ability to form stable mTORC1 and mTORC2 complexes, it is the acyl chain structure that makes the lipid recognizable for the protein in the membrane context. Our data show that besides structural properties of various PA species also some membrane features, e.g., the presence of other lipids such as CH, may exert a strong influence on the availability of the lipid for the protein to interact. Thus, some physiologically important consequences can be concluded while taking into consideration a few cellular aspects. First of all, it is already well known that the structure of PA depends on the synthesis pathways that within a cell are tightly spatiotemporally regulated. Namely, PA-producing enzymes are very often localized only at certain membrane compartments of a cell, they usually exhibit some preferences in terms of substrates, and their activity is driven by a variety of stimuli. Additionally, the intracellular distribution of CH is to a large degree inhomogeneous, which is reflected by, e.g., the steep gradient of this lipid along the secretory pathway. Phospholipases D (PLDs) are considered to produce predominantly PA species with one saturated and one unsaturated acyl chain, e.g., POPA. While taking into consideration strong interactions of POPA with POPC that may inhibit interactions of the former with mTOR and the effect of CH that completely abolishes such inhibition, a conclusion can be drawn that membrane recruitment of the protein takes place only at certain, precisely defined membrane sites of high CH and PA produced by PLD. Within the cellular plasma membrane, not only high content of CH would be able to modulate the presentation of PA but also other glycerophospholipids, such as phosphatidylethanolamine, which may change the net charge of PA via deprotonation and hydrogen bond formation. On the other hand, DPPA, which is usually linked to metabolic pathways driven towards the production of storage lipids (e.g., triacylglycerols), was shown to exert an inhibitory effect on mTORC complexes. It could be that this lipid is more intensively produced in selected cellular compartments (e.g., endoplasmic reticulum), thus sequestering PA-interacting proteins from certain locations and isolating them from some downstream effectors. Remarkably, DPPA–mTOR interactions are not as heavily influenced by the CH as in the case of POPA, although BLI binding curves show that the interactions have different characteristics. Thus, depending on the location within the cell and levels of various PA species, mTOR could be inactive or active as a part of either the mTORC1 or mTORC2 complex, each of which would be able to activate different downstream signaling pathways to a variable degree. Certainly, we are still far from providing a complete picture of molecular mechanisms that govern the selectivity and specificity of PA–mTOR interactions, as our study did not include the effect of pH, divalent ions, and other factors. This might be crucial in light of the fact that PA has been described as a cellular pH-biosensor and that the lipid is able to bind calcium ions in a pH-dependent manner, as discussed by us elsewhere [15]. However, we believe that the presented data allowed us to make a big step further in elucidating so far unknown modulatory mechanisms of lipid signaling.

## Figures and Tables

**Table 1:** Kinetic rate constants and affinities determined for the liposome/mTOR FRB domain interactions.

Liposomes	PA Concetration Range (µM)	Ka (1/Ms)	Kd (1/s)	KD (M)	KD2 (M)
POPC/CH/POPA	12–36	7.38 × 10^1^	2.53 × 10^−4^	3.43 × 10^−6^	
POPC/CH/SAPA	4–30	1.13 × 10^2^	3.92 × 10^−4^	3.46 × 10^−6^	
POPC/SAPA	8–36	7.26 × 10^1^	3.74 × 10^−4^	5.16 × 10^−6^	
POPC/CH/DPPA	12–36	1.96 × 10^1^	1.67 × 10^−4^	8.53 × 10^−6^	
POPC/DPPA	2–24	8.82 × 10^1^	3.06 × 10^−4^	3.47 × 10^−6^	4.4 × 10^−6^
		1.15 × 10^4^	5.07 × 10^−2^		

## Supplementary Materials

The following supporting information can be downloaded at: https://www.mdpi.com/article/10.3390/cells11010119/s1, Supplementary Materials and Methods, Figure S1: Size and zeta potential measurements, Figure S2: TLC of lipids extracted from liposomes, Figure S3: Characterization of mTOR FRB domain, Figure S4: Effect of cholesterol on binding preferences of FRB domain of human mTOR investigated via flotation assay, Figure S5: Representative confocal images of GUVs stained with DPPE-Rh in the presence of FRB-GFP, Figure S6: GFP binding to GUVs, Figure S7: Scouting of optimal concentration for immobilization of FRB domain on Ni-NTA sensors, Figure S8: Control of specific and non-specific binding of liposomes to Ni-NTA sensors tips.

## References

[1] Czogalla A., Grzybek M., Jones W., Coskun Ü. (2014). Validity and applicability of membrane model systems for studying interactions of peripheral membrane proteins with lipids. Biochim. Biophys. Acta-Mol. Cell Biol. Lipids.

[2] Lingwood D., Binnington B., Róg T., Vattulainen I., Grzybek M., Coskun Ü., Lingwood C.A., Simons K. (2011). Cholesterol modulates glycolipid conformation and receptor activity. Nat. Chem. Boil..

[3] MPizzuto M., Lonez C., Baroja-Mazo A., Martínez-Banaclocha H., Tourlomousis P., Gangloff M., Pelegrin P., Ruysschaert J.-M., Gay N.J., Bryant C.E. (2019). Saturation of acyl chains converts cardiolipin from an antagonist to an activator of Toll-like receptor-4. Cell. Mol. Life Sci..

[4] Van Galen J., Van Balkom B.W.M., Serrano R.L., Kaloyanova D., Eerland R., Stüven E., Helms J.B. (2010). Binding of GAPR-1 to negatively charged phospholipid membranes: Unusual binding characteristics to phosphatidylinositol. Mol. Membr. Biol..

[5] Weise C.F., Login F.H., Ho O., Gröbner G., Wolf-Watz H., Wolf-Watz M. (2014). Negatively charged lipid membranes promote a disorder-order transition in the Yersinia YscU protein. Biophys. J..

[6] Ouberai M.M., Wang J., Swann M.J., Galvagnion C., Guilliams T., Dobson C.M., Welland M.E. (2013). α-Synuclein senses lipid packing defects and induces lateral expansion of lipids leading to membrane remodeling. J. Biol. Chem..

[7] Drin G., Antonny B. (2010). Amphipathic helices and membrane curvature. FEBS Lett..

[8] Antonny B. (2011). Mechanisms of membrane curvature sensing. Annu. Rev. Biochem..

[9] Balla T. (2005). Inositol-lipid binding motifs: Signal integrators through protein-lipid and protein-protein interactions. J. Cell Sci..

[10] Sakane F., Hoshino F., Murakami C. (2020). New era of diacylglycerol kinase, phosphatidic acid and phosphatidic acid-binding protein. Int. J. Mol. Sci..

[11] Wang X., Devaiah S.P., Zhang W., Welti R. (2006). Signaling functions of phosphatidic acid. Prog. Lipid Res..

[12] Testerink C., Munnik T. (2005). Phosphatidic acid: A multifunctional stress signaling lipid in plants. Trends Plant Sci..

[13] Stace C.L., Ktistakis N.T. (2006). Phosphatidic acid- and phosphatidylserine-binding proteins. Biochim. Biophys. Acta-Mol. Cell Biol. Lipids.

[14] Tanguy E., Kassas N., Vitale N. (2018). Protein–Phospholipid Interaction Motifs: A Focus on Phosphatidic Acid. Biomolecules.

[15] Zegarlińska J., Piaścik M., Sikorski A.F., Czogalla A. (2018). Phosphatidic acid—A simple phospholipid with multiple faces. Acta Biochim. Pol..

[16] Buckland A.G., Wilton D.C. (2000). Anionic phospholipids, interfacial binding and the regulation of cell functions. Biochim. Biophys. Acta-Mol. Cell Biol. Lipids.

[17] Carman G.M., Henry S.A. (2013). Phosphatidic Acid Plays a Central Role in the Transcriptional Regulation of Glycerophospholipid Synthesis in *Saccharomyces cerevisiae*. J. Biol. Chem..

[18] Pleskot R., Li J., Žárský V., Potocký M., Staiger C.J. (2013). Regulation of cytoskeletal dynamics by phospholipase D and phosphatidic acid. Trends Plant Sci..

[19] Jang J.-H., Lee C.S., Hwang D., Ryu S.H. (2012). Understanding of the roles of phospholipase D and phosphatidic acid through their binding partners. Prog. Lipid Res..

[20] Burger K.N.J., Demel R.A., Schmid S.L., de Kruijff B. (2000). Dynamin is membrane-active: Lipid insertion is induced by phosphoinositides and phosphatidic acid. Biochemistry.

[21] daCosta C.J.B., Wagg I.D., McKay M.E., Baenziger J.E. (2004). Phosphatidic acid and phosphatidylserine have distinct structural and functional interactions with the nicotinic acetylcholine receptor. J. Biol. Chem..

[22] Kobayashi S., Hirakawa K., Horiuchi H., Fukuda R., Ohta A. (2013). Phosphatidic acid and phosphoinositides facilitate liposome association of Yas3p and potentiate derepression of ARE1 (alkane-responsive element one)-mediated transcription control. Fungal Genet. Biol..

[23] Horchani H., de Saint-Jean M., Barelli H.L.H.L., Antonny B. (2014). Interaction of the Spo20 membrane-sensor motif with phosphatidic acid and other anionic lipids, and influence of the membrane environment. PLoS ONE.

[24] Young B.P., Shin J.J.H., Orij R., Chao J.T., Li S.C., Guan X.L., Khong A., Jan E., Wenk M.R., Prinz W.A. (2010). Phosphatidic acid is a pH biosensor that links membrane biogenesis to metabolism. Science.

[25] Eaton J.M., Mullins G.R., Brindley D.N., Harris T.E. (2013). Phosphorylation of lipin 1 and charge on the phosphatidic acid head group control its phosphatidic acid phosphatase activity and membrane association. J. Biol. Chem..

[26] Ghosh S., Bell R.M. (1997). Regulation of raf-1 kinase by interaction with the lipid second messenger, phosphatidic acid. Biochem. Soc. Trans..

[27] Kooijman E.E., Burger K.N.J. (2009). Biophysics and function of phosphatidic acid: A molecular perspective. Biochim. Biophys. Acta-Mol. Cell Biol. Lipids.

[28] EKooijman E.E., Tieleman D.P., Testerink C., Munnik T., Rijkers D.T., Burger K.N., de Kruijff B. (2007). An electrostatic/hydrogen bond switch as the basis for the specific interaction of phosphatidic acid with proteins. J. Biol. Chem..

[29] Keith C.T., Schreiber S.L. (1995). PIK-related kinases: DNA repair, recombination, and cell cycle checkpoints. Science.

[30] Betz C., Hall M.N. (2013). Where is mTOR and what is it doing there?. J. Cell Biol..

[31] Laplante M., Sabatini D.M. (2012). mTOR Signaling in Growth Control and Disease. Cell.

[32] Kim L.C., Cook R.S., Chen J. (2017). mTORC1 and mTORC2 in cancer and the tumor microenvironment Laura. Oncogene.

[33] Oh W.J., Jacinto E. (2011). mTOR complex 2 signaling and functions. Cell Cycle.

[34] Ben-Sahra I., Howell J.J., Asara J.M., Manning B.D. (2013). Stimulation of de Novo Pyrimidine Synthesis by Growth Signaling Through mTOR and S6K1. Science.

[35] Hagiwara A., Cornu M., Cybulski N., Polak P., Betz C., Trapani F., Terracciano L., Heim M., Rüegg M.A., Hall M.N. (2012). Hepatic mTORC2 activates glycolysis and lipogenesis through Akt, glucokinase, and SREBP1c. Cell Metab..

[36] Sancak Y., Peterson T.R., Shaul Y.D., Lindquist R.A., Thoreen C.C., Bar-Peled L., Sabatini D.M. (2008). The rag GTPases bind raptor and mediate amino acid signaling to mTORC1. Science.

[37] Bar-Peled L., Chantranupong L., Cherniack A.D., Chen W.W., Ottina K.A., Grabiner B.C., Spear E.D., Carter S.L., Meyerson M., Sabatini D.M. (2013). A Tumor Suppressor Complex with GAP Activity for the Rag GTPases That Signal Amino Acid Sufficiency to mTORC1. Science.

[38] Ebner M., Sinkovics B., Szczygieł M., Ribeiro D.W., Yudushkin I. (2017). Localization of mTORC2 activity inside cells. J. Cell Biol..

[39] Foster D.A. (2009). Phosphatidic acid signaling to mTOR: Signals for the survival of human cancer cells. Biochim. Biophys. Acta.

[40] Ávila-Flores A., Santos T., Rincón E., Mérida I. (2005). Modulation of the mammalian target of rapamycin pathway by diacylglycerol kinase-produced phosphatidic acid. J. Biol. Chem..

[41] Gingras A., Raught B., Sonenberg N. (2001). Regulation of translation initiation by FRAP/mTOR. Genes Dev..

[42] Foster D.A. (2007). Regulation of mTOR by phosphatidic acid?. Cancer Res..

[43] You J.S., Frey J.W., Hornberger T.A. (2012). Mechanical Stimulation Induces mTOR Signaling via an ERK-Independent Mechanism: Implications for a Direct Activation of mTOR by Phosphatidic Acid. PLoS ONE.

[44] O’neil T.K., Duffy L.R., Frey J.W., Hornberger T.A. (2009). The role of phosphoinositide 3-kinase and phosphatidic acid in the regulation of mammalian target of rapamycin following eccentric contractions. J. Physiol..

[45] Fang Y., Vilella-Bach M., Bachmann R., Flanigan A., Chen J. (2001). Phosphatidic acid-mediated mitogenic activation of mTOR signaling. Science.

[46] Ballou L.M., Jiang Y.P., Du G., Frohman M.A., Lin R.Z. (2003). Ca^2+^- and phospholipase D-dependent and -independent pathways activate mTOR signaling. FEBS Lett..

[47] Ha S.H., Kim D.-H., Kim I.-S., Kim J.H., Lee M.N., Lee H.J., Kim J.H., Jang S.K., Suh P.-G., Ryu S.H. (2006). PLD2 forms a functional complex with mTOR/raptor to transduce mitogenic signals. Cell. Signal..

[48] Takahara T., Hara K., Yonezawa K., Sorimachi H., Maeda T. (2006). Nutrient-dependent multimerization of the mammalian target of rapamycin through the N-terminal HEAT repeat region. J. Biol. Chem..

[49] Veverka V., Crabbe T., Bird I., Lennie G., Muskett F.W., Taylor R.J., Carr M.D. (2008). Structural characterization of the interaction of mTOR with phosphatidic acid and a novel class of inhibitor: Compelling evidence for a central role of the FRB domain in small molecule-mediated regulation of mTOR. Oncogene.

[50] Toschi A., Lee E., Xu L., Garcia A., Gadir N., Foster D.A. (2009). Regulation of mTORC1 and mTORC2 Complex Assembly by Phosphatidic Acid: Competition with Rapamycin. Mol. Cell. Biol..

[51] Chen Y., Zheng Y., Foster D.A. (2003). Phospholipase D confers rapamycin resistance in human breast cancer cells. Oncogene.

[52] Jenkins G.M., Frohman M.A. (2005). Phospholipase D: A lipid centric review. Cell. Mol. Life Sci..

[53] Kassas N., Tanguy E., Thahouly T., Fouillen L., Heintz D., Chasserot-Golaz S., Bader M.F., Grant N.J., Vitale N. (2017). Comparative characterization of phosphatidic acid sensors and their localization during frustrated phagocytosis. J. Biol. Chem..

[54] Siddiqui R.A., Yang Y.C. (1995). Interleukin-11 induces phosphatidic acid formation and activates map kinase in mouse 3T3-L1 cells. Cell. Signal..

[55] Zhang C., Wendel A.A., Keogh M.R., Harris T.E., Chen J., Coleman R.A. (2012). Glycerolipid signals alter mTOR complex 2 (mTORC2) to diminish insulin signaling. Proc. Natl. Acad. Sci. USA.

[56] Menon D., Salloum D., Bernfeld E., Gorodetsky E., Akselrod A., Frias M.A., Sudderth J., Chen P.-H., DeBerardinis R., Foster D.A. (2017). Lipid sensing by mTOR complexes via de novo synthesis of phosphatidic acid. J. Biol. Chem..

[57] Foster D.A. (2013). Phosphatidic acid and lipid-sensing by mTOR. Trends Endocrinol. Metab..

[58] Jellqvist B., Hughes G.J., Pasquali C., Paquet N., Ravier F., Sanchez J.-C., Frutiger S., Hochstrasser D. (1993). The focusing positions of polypeptides in immobilized pH gradients can be predicted from their amino acid sequences. Electrophoresis.

[59] Rouser G., Fleischer S., Yamamoto A. (1970). Two Dimensional Thin Layer Chromatographic Separation of Polar Lipids and Determination of Phospholipids by Phosphorus Analysis of Spots. Lipids.

[60] Dimitrov D.S., Angelova M.I. (1988). Lipid swelling and liposome formation mediated by electric fields. J. Electroanal. Chem..

[61] Méléard P., Bagatolli L.A., Pott T. (2009). Giant Unilamellar Vesicle Electroformation. From Lipid Mixtures to Native Membranes Under Physiological Conditions. Methods Enzymol..

[62] Reeves J.P., Dowben R.M. (1969). Formation and properties of thin-walled phospholipid vesicles. J. Cell. Physiol..

[63] Tsumoto K., Matsuo H., Tomita M., Yoshimura T. (2009). Efficient formation of giant liposomes through the gentle hydration of phosphatidylcholine films doped with sugar. Colloids Surf. B Biointerfaces.

[64] Wallner J., Lhota G., Jeschek D., Mader A., Vorauer-uhl K. (2013). Application of Bio-Layer Interferometry for the analysis of protein/liposome interactions. J. Pharm. Biomed. Anal..

[65] Katsamba P., Navratilova I., Calderon-Cacia M., Fan L., Thornton K., Zhu M., Bos T.V., Forte C., Friend D., Laird-Offringa I. (2006). Kinetic analysis of a high-affinity antibody/antigen interaction performed by multiple Biacore users. Anal. Biochem..

[66] Contreras F.-X., Ernst A., Haberkant P., Björkholm P., Lindahl E., Gönen B., Tischer C., Elofsson A., von Heijne G., Thiele C. (2012). Molecular recognition of a single sphingolipid species by a protein’s transmembrane domain. Nature.

[67] Shulga Y.V., Topham M.K., Epand R.M. (2011). Regulation and functions of diacylglycerol kinases. Chem. Rev..

[68] Drabik D., Czogalla A. (2021). Simple Does Not Mean Trivial: Behavior of Phosphatidic Acid in Lipid Mono- and Bilayers. Int. J. Mol. Sci..

[69] SAntollini S., Soto M.A., de Romanelli I.B., Gutiérrez-Merino C., Sotomayor P., Barrantes F.J. (1996). Physical state of bulk and protein-associated lipid in nicotinic acetylcholine receptor-rich membrane studied by laurdan generalized polarization and fluorescence energy transfer. Biophys. J..

[70] Tattrie N.H., Bennett J.R., Cyr R. (1968). Maximum and minimum values for lecithin classes from various biological sources. Can. J. Biochem..

[71] Sezgin E., Schwille P. (2012). Model membrane platforms to study protein-membrane interactions. Mol. Membr. Biol..

[72] Macháň R., Hof M. (2010). Lipid diffusion in planar membranes investigated by fluorescence correlation spectroscopy. Biochim. Biophys. Acta-Biomembr..

[73] Kulig W., Korolainen H., Zatorska M., Kwolek U., Kepczynski M. (2019). Complex Behavior of Phosphatidylcholine—Phosphatidic Acid Bilayers and Monolayers: Effect of Acyl Chain Unsaturation. Langmuir.

[74] Cambrea L.R., Haque F., Schieler J.L., Rochet J.-C., Hovis J.S. (2007). Effect of Ions on the Organization of Phosphatidylcholine/Phosphatidic Acid Bilayers. Biophys. J..

[75] Incardona J.P., Eaton S. (2000). Cholesterol in signal transduction. Curr. Opin. Cell Biol..

[76] Ikonen E. (2008). Cellular cholesterol trafficking and compartmentalization. Nat. Rev. Mol. Cell Biol..

[77] Ouweneel A.B., Thomas M.J., Sorci-Thomas M.G. (2020). The ins and outs of lipid rafts: Functions in intracellular cholesterol homeostasis, microparticles, and cell membranes. J. Lipid Res..

[78] Hryniewicz-Jankowska A., Augoff K., Sikorski A.F. (2019). Highlight article: The role of cholesterol and cholesterol-driven membrane raft domains in prostate cancer. Exp. Biol. Med..

[79] Drabik D., Gavutis M., Valiokas R.N., Ulčinas A.R. (2020). Determination of the Mechanical Properties of Model Lipid Bilayers Using Atomic Force Microscopy Indentation. Langmuir.

[80] Barros M., Heinrich F., Datta S.A.K., Rein A., Karageorgos I., Nanda H., Lösche M. (2016). Membrane Binding of HIV-1 Matrix Protein: Dependence on Bilayer Composition and Protein Lipidation. J. Virol..

[81] Henderson J., Iyengar N.S., Lam K.L.H., Maldonado E., Suwatthee T., Roy I., Waring A.J., Lee K.Y.C. (2019). Beyond electrostatics: Antimicrobial peptide selectivity and the influence of cholesterol-mediated fluidity and lipid chain length on protegrin-1 activity. Biochim. Biophys. Acta-Biomembr..

[82] Heiner A.L., Gibbons E., Fairbourn J.L., Gonzalez L.J., McLemore C.O., Brueseke T.J., Judd A.M., Bell J.D. (2008). Effects of cholesterol on physical properties of human erythrocyte membranes: Impact on susceptibility to hydrolysis by secretory phospholipase A2. Biophys. J..

[83] Warnock D.E., Roberts C., Lutz M.S., Blackburn W.A., Young W.W., Baenziger J.U. (1993). Determination of plasma membrane lipid mass and composition in cultured Chinese hamster ovary cells using high gradient magnetic affinity chromatography. J. Biol. Chem..

[84] Maxfield F.R., Menon A.K. (2006). Intracellular sterol transport and distribution. Curr. Opin. Cell Biol..

[85] Martello A., Platt F.M., Eden E.R. (2020). Staying in touch with the endocytic network: The importance of contacts for cholesterol transport. Traffic.

[86] Litvinov D.Y., Savushkin E.V., Dergunov A.D. (2018). Intracellular and Plasma Membrane Events in Cholesterol Transport and Homeostasis. J. Lipids..

[87] Camargo D.C.R., Link N.M., Dames S.A. (2012). The FKBP-rapamycin binding domain of human tor undergoes strong conformational changes in the presence of membrane mimetics with and without the regulator phosphatidic acid. Biochemistry..

[88] Capelluto D.G., Zhao X., Lucas A., Lemkul J.A., Xiao S., Fu X., Sun F., Bevan D.R., Finkielstein C.V. (2014). Biophysical and molecular-dynamics studies of phosphatidic acid binding by the Dvl-2 DEP domain. Biophys. J..

[89] Eaton J.M., Takkellapati S., Lawrence R.T., McQueeney K.E., Boroda S., Mullins G.R., Sherwood S.G., Finck B.N., Villen J., Harris T.E. (2014). Lipin 2 binds phosphatidic acid by the electrostatic hydrogen bond switch mechanism independent of phosphorylation. J. Biol. Chem..

[90] Boughter C.T., Monje-Galvan V., Im W., Klauda J.B. (2016). Influence of Cholesterol on Phospholipid Bilayer Structure and Dynamics. J. Phys. Chem. B.

[91] Barbour R., Bova M.P. (2012). Combining label-free technologies: Discovery in strength. Bioanalysis.

[92] DMyszka G., Jonsen M.D., Graves B.J. (1998). Equilibrium analysis of high affinity interactions using BIACORE. Anal. Biochem..

[93] Abdiche Y., Malashock D., Pinkerton A., Pons J. (2008). Determining kinetics and affinities of protein interactions using a parallel real-time label-free biosensor, the Octet. Anal. Biochem..

[94] Rich R.L., Myszka D.G. (2009). Extracting kinetic rate constants from binding responses. Label-Free Biosens. Tech. Appl..

[95] Wallner J., Lhota G., Schosserer M., Vorauer-Uhl K. (2017). An approach for liposome immobilization using sterically stabilized micelles (SSMs) as a precursor for bio-layer interferometry-based interaction studies. Colloids Surf. B Biointerfaces.

