# Supplementary material for: Selectivity of mTOR-Phosphatidic Acid Interactions Is Driven by Acyl Chain Structure and Cholesterol
Source: Cells. 2021 Dec 30;11(1):119. doi: 10.3390/cells11010119 (PMC8750377; doi:10.3390/cells11010119)
Supplement: Supplementary file 1 [file cells-11-00119-s001.zip › cells-1509154-supplementary.pdf]

Supplementary Materials: Selectivity of mTOR - phosphatidic acid interactions is driven by acyl chain structure and cholesterol

Jolanta Żelasko, Aleksander Czogalla

Department of Cytobiochemistry, Faculty of Biotechnology, F. Joliot-Curie 14a, 50-383 Wroclaw, Poland; aleksander.czogalla@uwr.edu.pl

1. Size and zeta potential measurements

Size and zeta potential of LUVs were determined using a ZetaSizer Nano ZS. For size and zeta-potential measurements LUVs were diluted to a final concentration of lipids of 0.1 mg/mL in SLB buffer (for size) or Milli-Q water (for zeta-potential). Importantly, the buffer was filtered (PVDF, 0.45 μm, Millipore) prior to use.

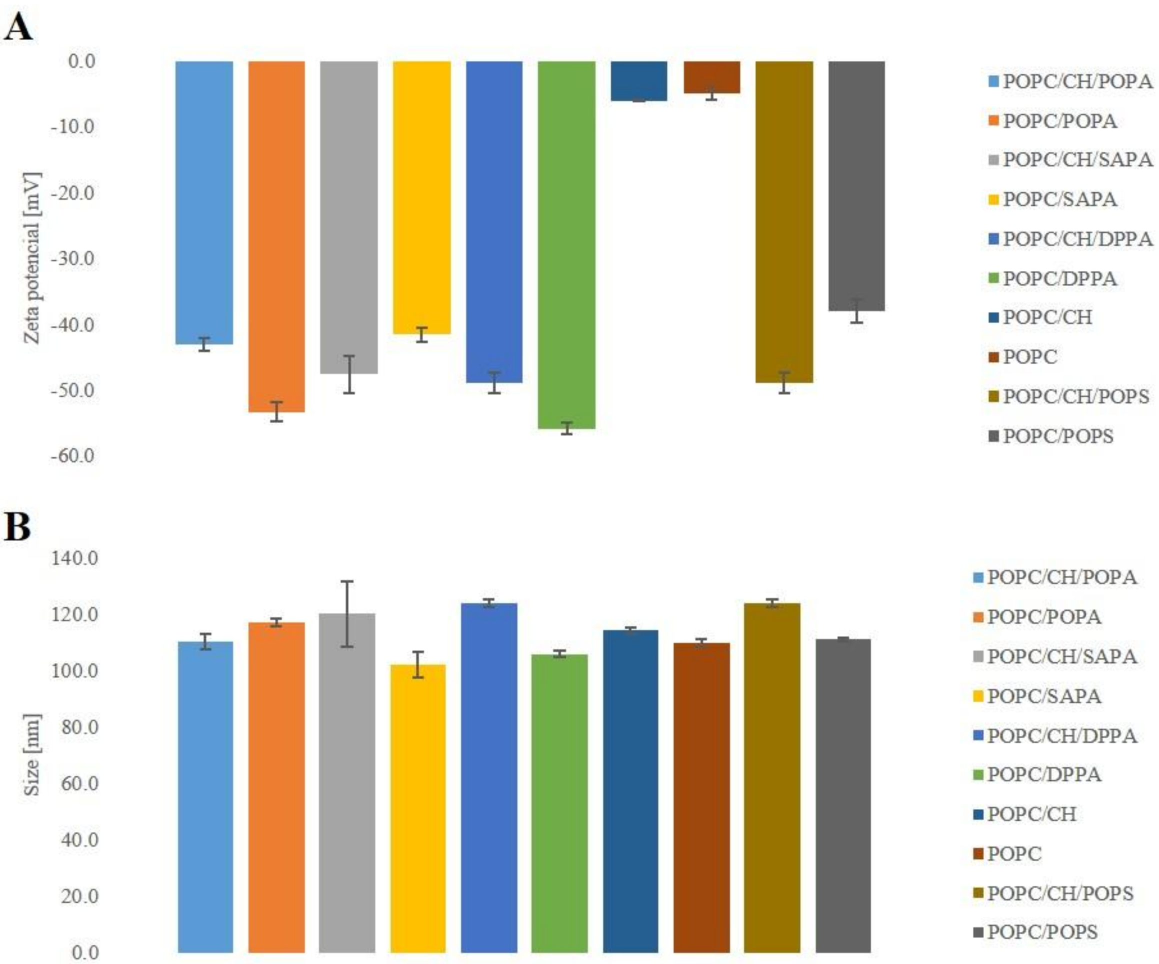

PDI	0.05	0.06	0.08	0.08	0.07	0.1	0.06	0.08	0.05	0.08

Figure S1. Quality controls of LUVs; A) Zeta potential of LUVs; the data represent the average from three independent experiments. Error bars are standard deviation values (SD); B) Size distribution of LUVs with polydispersity index (PDI) indicated below in table; the data represent the average from three independent experiments. Error bars are standard deviation values (SD).

2. TLC

Quality control of LUVs was performed with thin layer chromatography. Lipids were extracted from liposomes by mixing 100 µl of 1 mg/mL with 150 µl of buffer and 500 µl of solvent (chloroform/methanol/acetone/HCl (1N) in the proportion 2/1/0.5/0.1) to a final volume of 750 µl, vortexing and incubating on ice for 20 min, then centrifuged (1000 g) for 5 min. Afterwards, the upper phase was discarded and the organic phase dried. Next, lipids were dissolved in 20 µl of solvent (chloroform/methanol/acetone/HCl (1N) 2/1/0.5/0.1 v/v), vortexed and loaded on plates, previously washed with solvent. Plates were run in the solvent system: chloroform/acetone/methanol/acetic acid/water (46/17/15/14/8, v/v). The solvent was allowed to migrate up to 1 cm from the top edge of a 20 cm TLC plate, dried and the lipid spots were identified under a UV lamp after spraying a primuline solution (5 mg in 100 mL of acetone/water, 80/20, v/v).

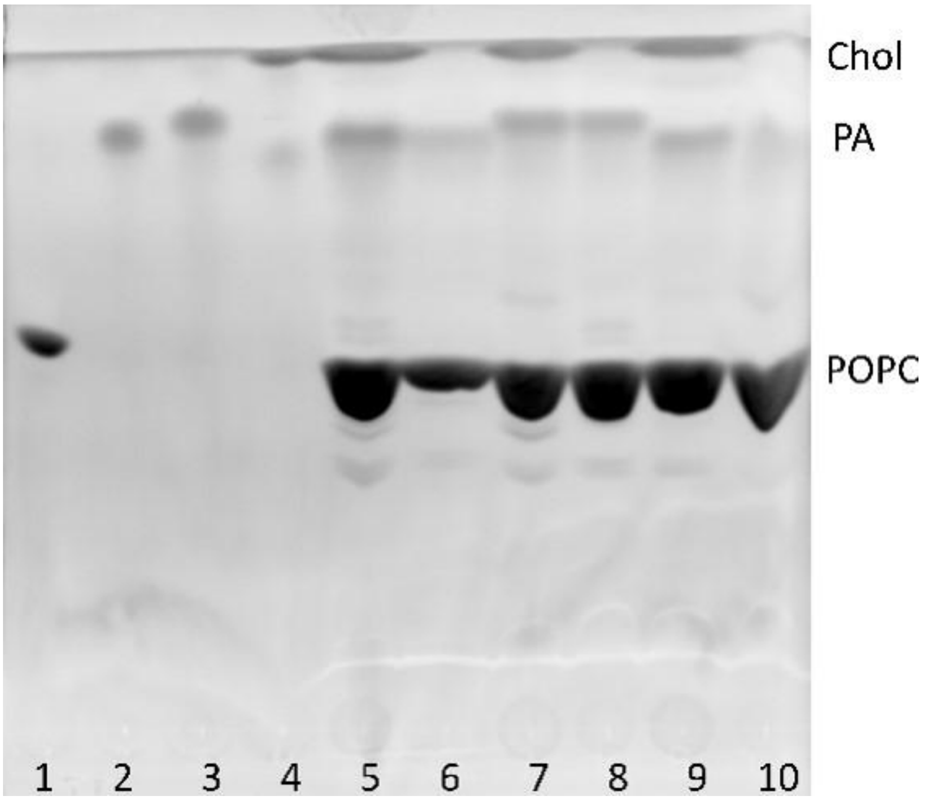

Figure S2. TLC of lipids extracted from liposomes. Sample were loaded in the following order: 1–4 standards: POPC, POPA, SAPA, DPPA+ Chol, 5 – POPC/CH/POPA, 6 – POPC/POPA, 7 – POPC/CH/SAPA, 8 – POPC/SAPA, 9 – POPC/CH/DPPA, 10 – POPC/DPPA.

3. Circular dichroism and size-exclusion chromatography of FRB domain

Circular dichroism measurements were performed using a spectropolarimeter J-1500 (Jasco) with 0.1 cm path length cuvette. Circular dichroism spectra of mTOR FRB-His tag domain were measured at 20 °C and the melting curve was measured in a temperature range of 10-65 °C. Protein samples for all measurements were diluted at a concentration of 0.15 mg/mL in PBS buffer. The data were normalized to mean residue ellipticity using the following equation (where $\boldsymbol{n}$ is the number of peptide bonds in the protein and $\boldsymbol{ellipticity}$ is the raw data from the instrument):

$$mean\ \ residue\ \ ellipticity\ \ [mdeg \times cm^2 \times dmol^{-1}] = \frac{ellipticity\ \ [mdeg]}{10 \times pathlength\ \ [cm] \times concentration\ \ \left[\frac{mol}{L}\right] \times n}$$

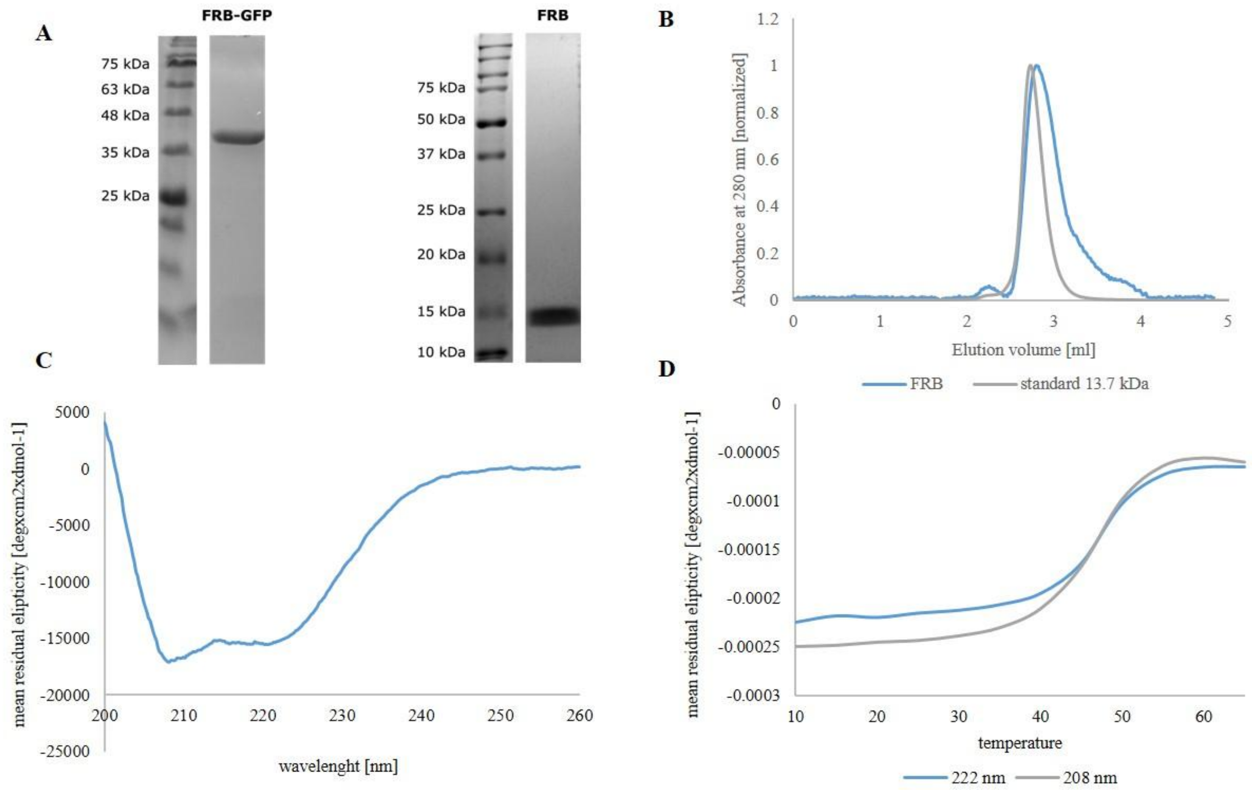

Figure S3. Characterization of mTOR FRB domain. **A)** SDS-PAGE of bacterially expressed and purified FRB GFP and FRB domains in 12% polyacrylamide gel. **B)** Elution profile of purified mTOR FRB domain on a Superdex 200 5/150 size-exclusion column (flow rate 0.3 mL/min). Ribonuclease A used as a standard. **C)** Circular dichroism spectra of FRB-His tag domain measured at 20 °C and **D)** melting curves registered at 222 nm (blue) and 208 nm (grey).

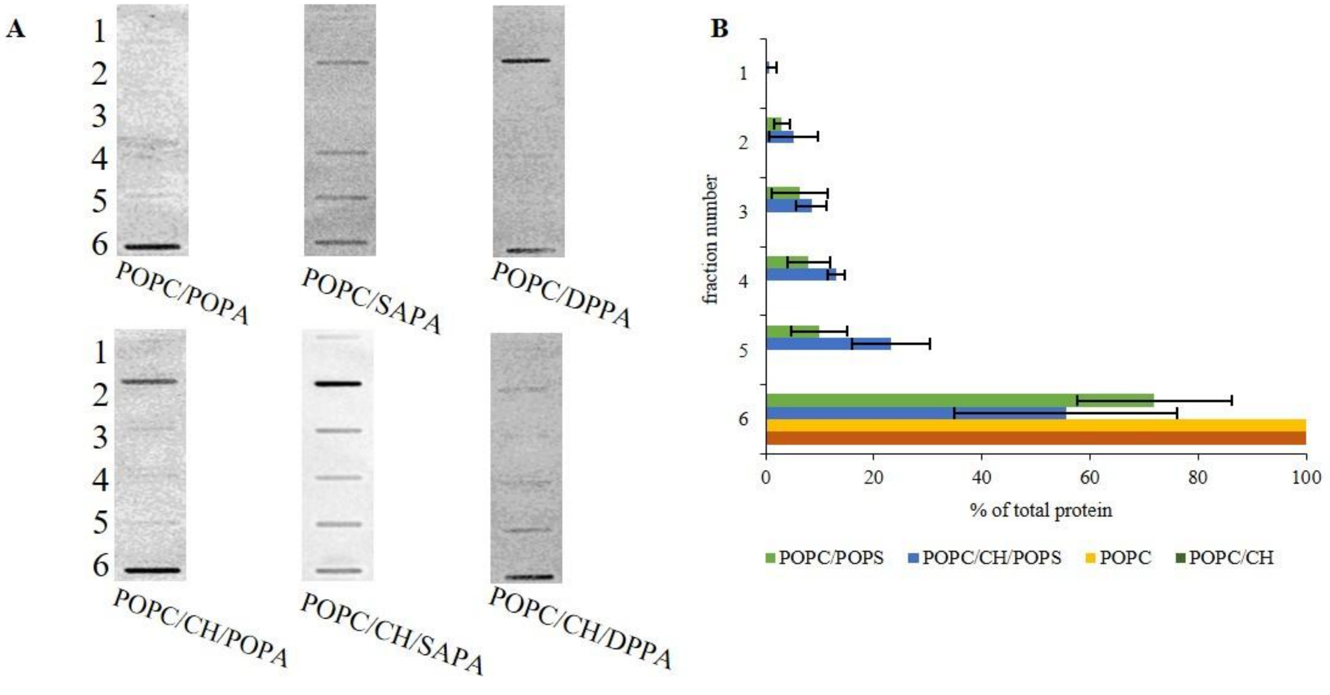

Figure S4. Effect of cholesterol on binding preferences of FRB domain of human mTOR investigated via flotation assay. A) Representative examples of original Dot-Blots of fractions collected after flotation corresponding to the data presented in Figure 2A **B)** Effect of cholesterol on binding preferences of FRB domain of human mTOR. FRB domain of mTOR protein content analyzed by dot-blot/densitometry of fractions collected after flotation. It can be observed that for all tested phospholipids no or very weak binding occurs. Error bars are standard deviations and are presented for three independent experiments.

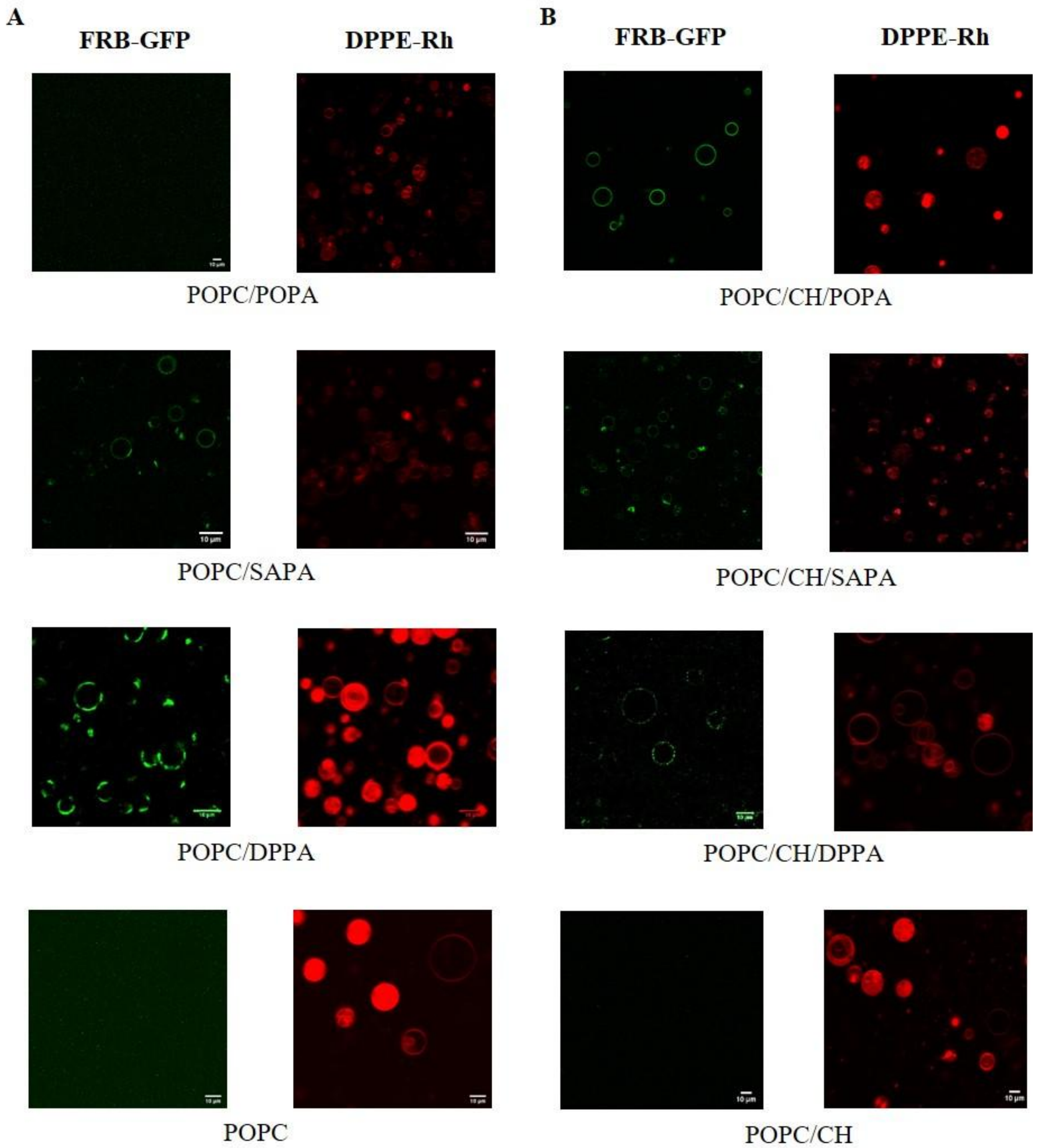

Figure S5. Representative confocal images of GUVs stained with DPPE-Rh (red) in the presence of FRB-GFP (green). Scale bars correspond to 10 µm. A) GUVs with three molecular species of PA (POPC/PA; 90/10 mol%) and POPC as a control. B) GUVs with addition of CH (35 mol%) to corresponding lipid mixtures shown in (A).

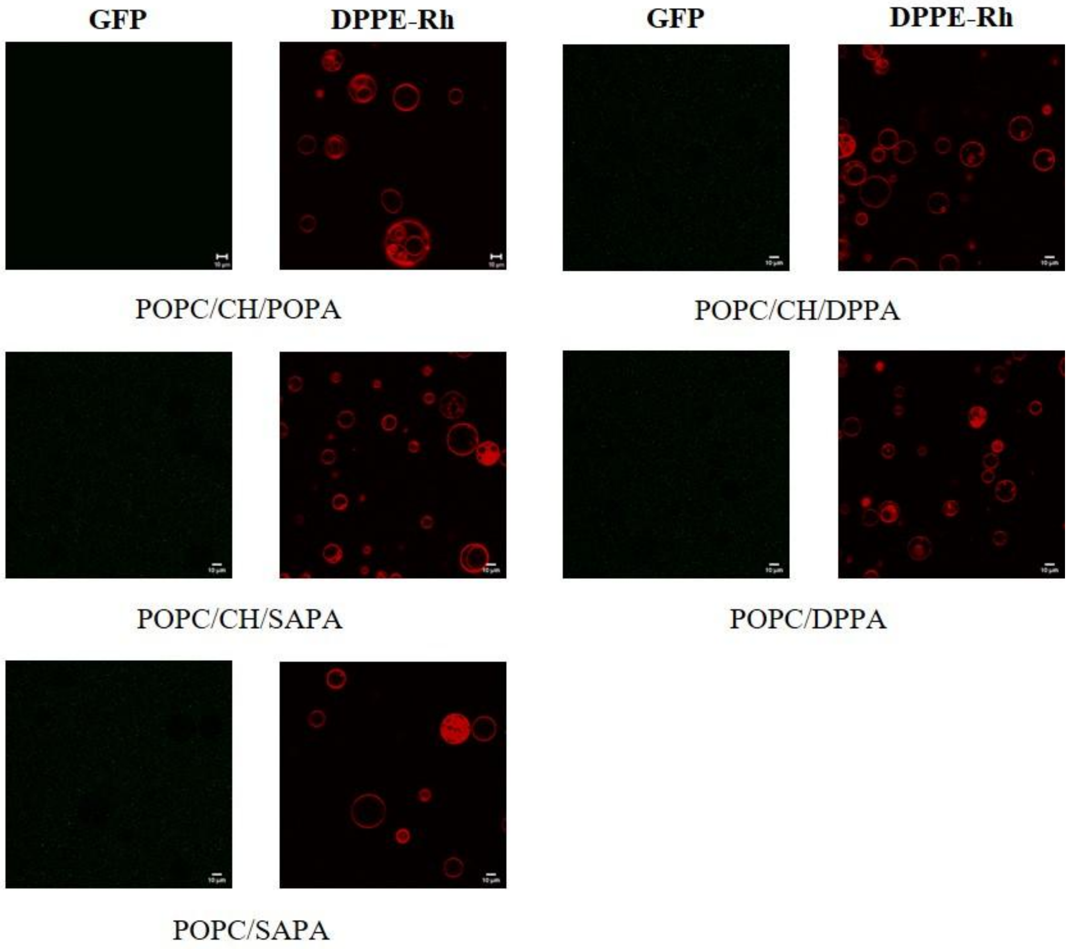

Figure S6. GFP (green) binding to GUVs (stained with DPPE-Rh, red). Scale bars correspond to 10 μm. Results are presented for GUVs for which interaction with FRB-GFP was observed.

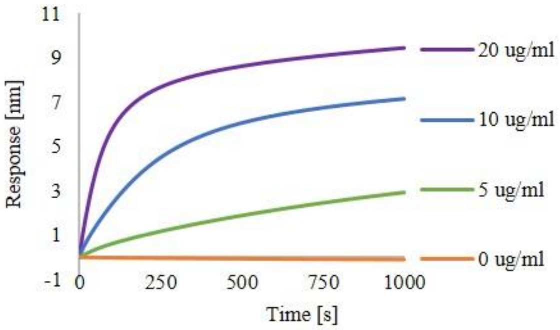

Figure S7. Scouting of optimal concentration for immobilization of FRB domain on Ni-NTA sensors. The loading step with the mTOR FRB domain in concentration range of 0–20 μg/mL.

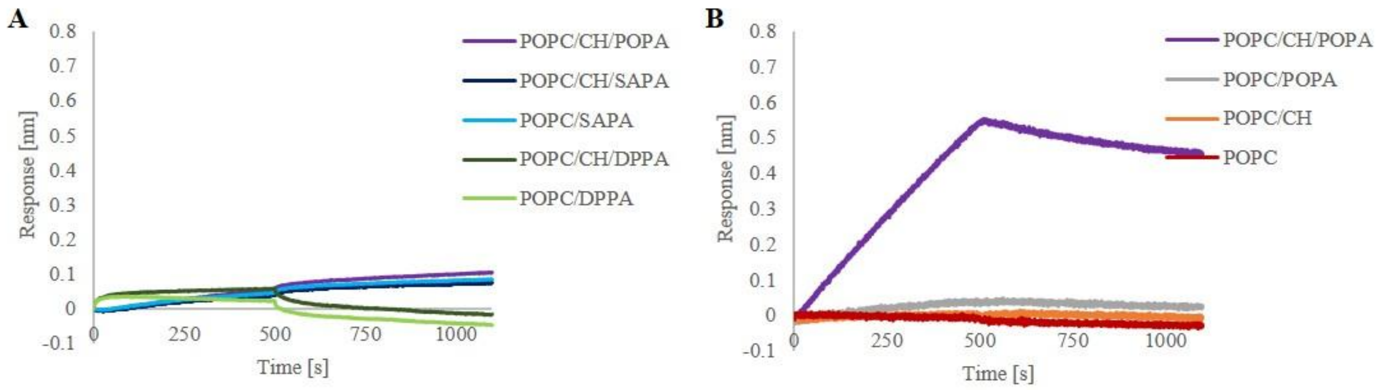

Figure S8. Control of specific and non-specific binding of liposomes to Ni-NTA sensors tips. A) Raw association/dissociation curves represent non-specific binding of PA-containing liposomes to sensor tips in absence of protein. B) YES/NO binding assays of liposomes for which previously we did not observe interaction; as a reference, the association/dissociation curve of POPC/CH/POPA was added (gray). In both panels the Y axis was adjusted to the value used in **Figure 3.** Liposomes were applied at PA concentration of 36 μM.
